# Improving Familial Hypercholesterolemia Index Case Detection: Sequential Active Screening from Centralized Analytical Data

**DOI:** 10.3390/jcm10040749

**Published:** 2021-02-13

**Authors:** Fernando Sabatel-Pérez, Joaquín Sánchez-Prieto, Víctor Manuel Becerra-Muñoz, Juan Horacio Alonso-Briales, Pedro Mata, Luis Rodríguez-Padial

**Affiliations:** 1 Department of Cardiology, Complejo Hospitalario Universitario de Toledo, 45004 Toledo, Spain; joaquinsanchezprieto@gmail.com (J.S.-P.); lrpadial@gmail.com (L.R.-P.); 2 Unidad de Gestión Clínica Área del Corazón, Instituto de Investigación Biomédica de Málaga (IBIMA), Hospital Universitario Virgen de la Victoria de Málaga, Universidad de Málaga (UMA), Centro de Investigación Biomédica en Red de Enfermedades Cardiovasculares (CIBERCV), 29010 Málaga, Spain; vmbecerram@gmail.com (V.M.B.-M.); juanhalonso62@gmail.com (J.H.A.-B.); 3 Fundación Hipercolesterolemia Familiar, 28010 Madrid, Spain; pmata@colesterolfamiliar.org

**Keywords:** familial hypercholesterolemia, genetic screening, atherosclerosis prevention, early detection

## Abstract

The majority of familial hypercholesterolemia index cases (FH-IC) remain underdiagnosed and undertreated because there are no well-defined strategies for the universal detection of FH. The aim of this study was to evaluate the diagnostic yield of an active screening for FH-IC based on centralized analytical data. From 2016 to 2019, a clinical screening of FH was performed on 469 subjects with severe hypercholesterolemia (low-density lipoprotein cholesterol ≥220 mg/dL), applying the Dutch Lipid Clinic Network (DLCN) criteria. All patients with a DLCN ≥ 6 were genetically tested, as were 10 patients with a DLCN of 3–5 points to compare the diagnostic yield between the two groups. FH was genetically confirmed in 57 of the 84 patients with DLCN ≥ 6, with a genetic diagnosis rate of 67.9% and an overall prevalence of 12.2% (95% confidence interval: 9.3% to 15.5%). Before inclusion in the study, only 36.8% (*n* = 21) of the patients with the FH mutation had been clinically diagnosed with FH; after genetic screening, FH detection increased 2.3-fold (*p* < 0.001). The sequential, active screening strategy for FH-IC increases the diagnostic yield for FH with a rational use of the available resources, which may facilitate the implementation of FH universal and family-based cascade screening strategies.

## 1. Introduction

Familial hypercholesterolemia (FH) is the genetic disorder most frequently associated with premature atherosclerotic cardiovascular disease (ASCVD) [1] due to lifelong elevated levels of low-density lipoprotein cholesterol (LDL-C). Its prevalence in the general population ranges from 1 in every 200 to 300 individuals [2,3,4,5]. It has an autosomal dominant transmission pattern whose causative mutations are mainly in the *LDL receptor* gene *(LDL-R)* and, less frequently, mutations in the *apolipoprotein B (APOB)* and *proprotein convertase subtilisin/kexin type 9 (PCSK9)* genes.

Clinical diagnosis of FH is based on clinical and analytical criteria, of which the most widely used and recommended are the Dutch Lipid Clinic Network (DLCN) criteria [6,7], as they have been validated with genetic diagnosis [8,9]. Genetic study is the gold standard for confirming FH, and it is necessary once the clinical diagnosis is probable or definite [10]. FH is markedly related to the development of ASCVD [11,12] with up to 22-fold increased risk compared to the general population [13,14,15]. Therefore, early detection is essential to both start and optimize treatment, which drastically reduces the risk of ASCVD [16,17,18,19], and perform family-based cascade screening. Despite international recommendations in FH clinical and genetic diagnosis [10], the main problem is the lack of clearly defined screening strategies for the identification of FH index cases in the general population, as well as insufficient genetic test availability [20]. This is why diagnosis is currently performed either after the development of clinical ASCVD or after the fortuitous detection of abnormally high LDL-C levels. Consequently, the majority of patients with FH remain underdiagnosed and undertreated [21].

The aim of this study was to evaluate the genetic diagnostic yield of a sequential, active screening of FH index cases in a population with severe hypercholesterolemia (HCL), based on centralized analytical data, to perform clinical and genetic family-based cascade screening. This screening strategy can contribute to changing how this severe disease is currently detected in Spain.

## 2. Materials and Methods

### 2.1. Population Study and Clinical Diagnostic Criteria for Familial Hypercholesterolemia

We conducted a selective, single-phase, active screening of patients aged ≥18 years with severe HCL, defined as total cholesterol ≥ 290 mg/dL, LDL-C ≥ 220 mg/dL, and triglycerides (TG) ≤ 200 mg/dL, the recommended values at which to start FH screening [7]. Analytical records from both primary and hospital care settings of the whole health area, which are centralized in the Biochemical Laboratory of the Hospital Complex, were reviewed. All samples with the stated profile for the years 2013, 2014, and 2015 were selected. Health professionals not connected with our study had previously requested the biochemical analyses for a variety of reasons. Blood samples were extracted after fasting, and LDL-C levels were calculated using the Friedewald formula [22]. A sample of saliva was taken for genetic testing. The lipid-lowering treatment (LLT) of each patient and their adherence to it was analyzed before inclusion in the study, with LDL-C levels adjusted according to Masana’s correction [23], as long as the patient was correctly following the treatment. Maximal lipid-lowering therapy was defined as any LLT that reduces LDL-C levels ≥50% [24].

Demographic and clinical variables, age, classic cardiovascular risk factors, and physical examination were included. We considered ASVCD whenever medical records included a clinical diagnosis of non-thromboembolic stroke, peripheral artery disease (PAD), or ischemic heart disease, both stable and acute coronary syndrome (ACS).

All patients consecutively underwent a medical assessment from December 2016 to March 2019 and were classified following DLCN criteria [25]. These criteria assign a score according to LDL-C levels, family history of HCL in children and/or parents (of the index case), history of premature ASCVD in the index case and/or family members, and the presence of tendon xanthoma or corneal arcus in a person <45 years (Table S1). Consistent with the score obtained, the diagnosis of FH may be possible (3–5 points), probable (6–7 points), or definite (≥8 points). We excluded subjects with a secondary cause of HCL (uncontrolled thyroid condition, HIV on antiretroviral therapy, nephrotic syndrome, end stage chronic kidney disease, uncontrolled diabetes, combined hyperlipidemia, and hepatobiliary diseases), the deceased, subjects with a diagnosis of terminal illness, anyone who refused to participate in the study, and those who were impossible to contact.

This study was approved by the Ethics Committee of the Complejo Hospitalario de Toledo (16 June 2016) and conducted in compliance with the Declaration of Helsinki recommendations for medical research involving human subjects. All of the patients who participated signed informed consent for the purposes contemplated in legislation current at the time the study began.

### 2.2. Genetic Testing

The genetic testing was performed using the Lipid inCode^®^ test (GENinCode, Barcelona, Spain), which is based on next-generation sequencing (NGS). Subsequently, ultrasequencing was used to detect abnormalities in the DNA sequence of the promoter regions that codify and form the exon-intron boundaries of 7 genes: *LDLR, APOB, PCSK9, APOE, STAP1, LDLRAP1,* and *LIPA*. The laboratory used the Gendicall 3.0, a bioinformatics tool developed by GENinCode (Gendicall 3.0, GENinCode, Rambla d’Ègara, Terrassa, Spain), to analyze the obtained results. The Lipid inCode^®^ test methodology has previously been used in Spanish studies [26]. Variant annotation was based on the Human Genome Variation Society standard [27] using isoforms from the Reference Sequence (REFSEQ) database, Ensembl 81 source (www.ensembl.org accessed on 10 February 2021). Variant interpretation and pathogenicity classification followed information from the Gendiag.exe database of genetic variants, adhering to the rules published by the American College of Medical Genetics and Genomics (ACMG) [28]. Consistent with ACMG criteria, clinically relevant variants were classified as pathogenic (class I), likely pathogenic (class II), and variants of unknown significance (class III). Since *STAP1* is very unlikely to be a causative FH gene, variants in this gene were not considered [29].

### 2.3. Statistical Analysis

Quantitative variables are presented as a mean ± standard deviation, whereas qualitative variables are given as numbers (%). The Kolmogorov–Smirnov test was used to check the normal distribution of variables. We used standardized effect size measures for baseline characteristics, estimated Cohen’s D for quantitative variables, and calculated the odds ratio for qualitative variables, with the respective 95% confidence intervals. Pearson’s chi-square test and Fisher’s exact test were used to compare the ASCVD percentages between FH mutation and no FH mutation in the DLCN ≥ 6 group, as well as the diagnostic rate before and after the screening. Odds ratios were estimated to assess the probability of a positive genetic result according to the clinical diagnosis of FH.

The statistical significance level was established at *p* < 0.05. All calculations were performed using the IBM SPSS Statistics program, version 25.0 (Chicago, IL, USA).

## 3. Results

### 3.1. Clinical Characteristics of the Studied Population

**Figure 1:**
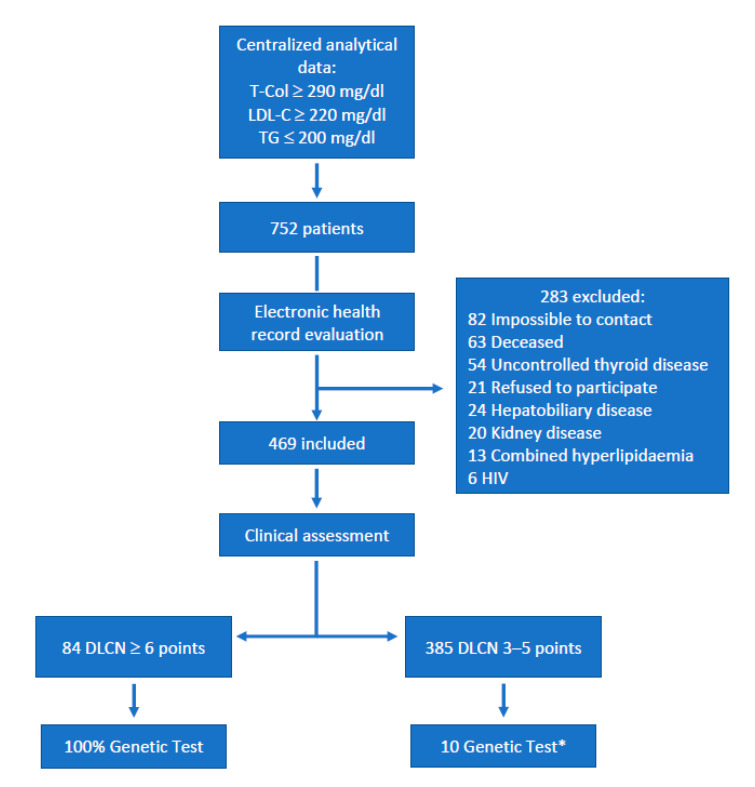
Patient selection: Flowchart showing sequential steps during patient selection. * Selected by means of consecutive sampling. T-Col: Total cholesterol. DLCN: Dutch Lipid Clinic Network. HIV: Human immunodeficiency virus. LDL-C: low-density lipoprotein cholesterol. TG: triglycerides.

A total of 752 subjects were evaluated, of which 283 were excluded. Therefore, 469 subjects were included and medically assessed. Of them, 385 subjects (82.1%) received a diagnosis of possible FH, whereas 84 (17.9%) had a diagnosis of probable or definite FH. Genetic tests were performed in all patients with DLCN ≥ 6 as well as 10 patients with DLCN 3–5, selected by means of consecutive sampling to compare the yield of genetic testing between both groups (Figure 1).

Of the 469 subjects, the proportion of men was 40.7%, and the mean age was 53.2 ± 12.8 years. The mean levels of total cholesterol and LDL-C were 331.7 ± 48.3 and 246.8 ± 38.2 mg/dL, respectively. Regarding the treatment, 73.4% of the subjects were on LLT; however, only 23.2% of this treatment was maximal lipid-lowering therapy. Concerning cardiovascular events, 5.5% of the patients had developed ASCVD, which was premature in 42.3% of them. Patients with DLCN ≥ 6, compared with the DLCN 3–5 group, were younger, had higher levels of total cholesterol and LDL-C, and had higher proportions of active or past smoking, LLT, and a family history of premature ASCVD and hypercholesterolemia (Table 1). Finally, the proportion of ASCVD was markedly higher in the DLCN ≥ 6 group, both globally and premature, and these findings were particularly notable in the group with FH mutation who had a global rate of 19.3%, and within this group, events occurred at a premature age in 63.6%. Coronary artery disease was the most frequent ASCVD in DLCN ≥ 6 with FH mutation (Table 2).

### 3.2. Genetic FH Diagnosis

**Figure 2:**
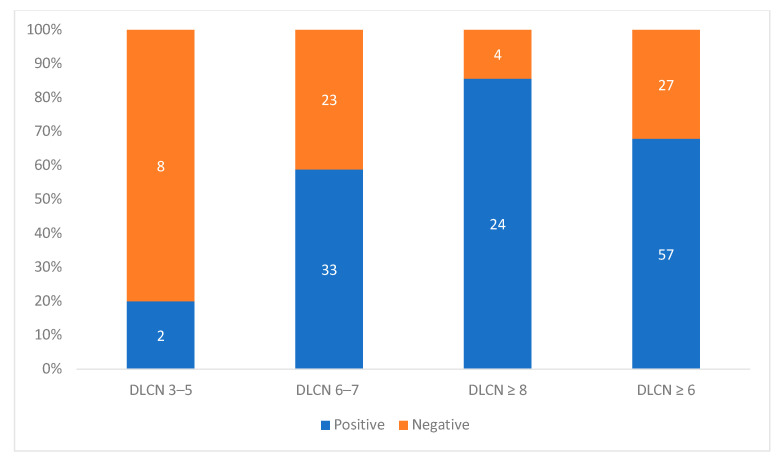
The positive and negative rates in the genetic study. Bar graph presenting the percentages of genetic diagnosis according to DLCN criteria. DLCN: Dutch Lipid Clinic Network.

Among the 84 subjects with DLCN ≥ 6, clinical FH diagnosis was confirmed with genetic testing in 57 (12.2% of the 469 included, 67.9% of those genetically studied). Differentiating by FH clinical diagnosis, 33 (58.9%) of the 56 patients with probable FH and 24 (85.7%) of the 28 patients with a definite FH (≥8 points) were genetically confirmed. The rate of genetic confirmation among patients with a clinical diagnosis of possible FH was 20%. The odds ratio for detection mutations in the DLCN ≥ 6 group compared to the DLCN 3–5 group was 8.44 (95% CI (1.68 to 42.49); *p* = 0.005; Figure 2, Table 3).

**Figure 3:**
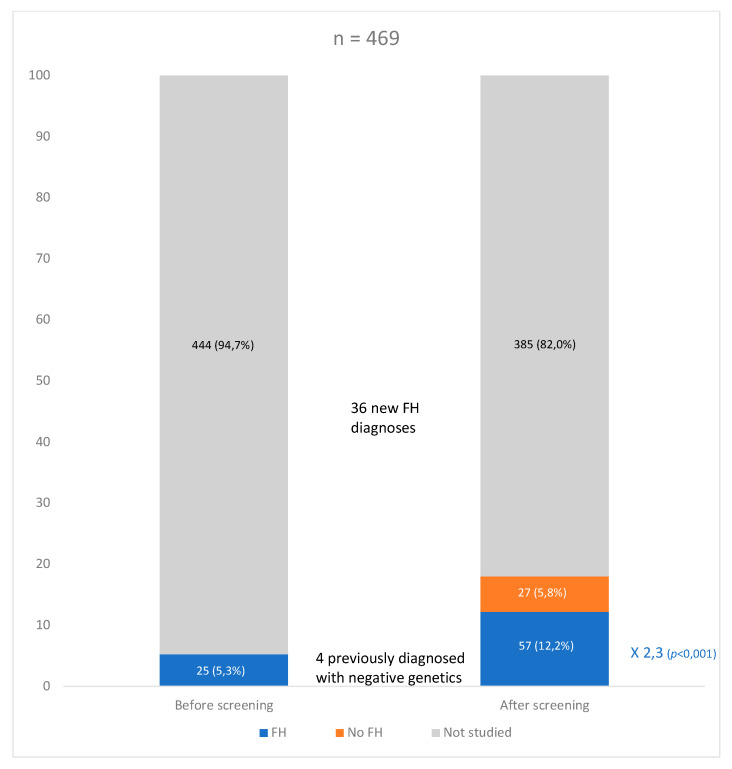
Diagnostic percentage of familial hypercholesterolemia, before and after screening, Bar graph where we can appreciate the percentage of diagnosis of familial hypercholesterolemia (FH) in the DLCN **≥** 6 group before carrying out the screening strategy (25 patients, 5.3%; although 4, 0.9%, had negative genetics and 21, 4.4%, were positive) and after the strategy (57, 12.2%, genetically confirmed with 2.3-fold increase in FH diagnosis, *p* < 0.001). The 10 patients with DLCN 3–5 and genetic study were counted as not studied. DLCN: Dutch Lipid Clinic Network.

Before their inclusion in the study, only 21 out of the 57 patients (36.8%) with genetic diagnosis had been clinically diagnosed with FH. Following the study strategy used in this research, the real detection rate in the cohort increased from 5.3% to at least 12.2% (2.3-fold increase; *p* < 0.001); it was also possible to reclassify four patients with no FH mutation who had been clinically diagnosed with FH before the present study (Figure 3).

The most frequent mutation was in the *LDLR* gene (92.9%), followed by *APOB* (5.3%) and *APOE* (1.8%). In our study, a mutation of the *LDLR* or *APOB* genes explained 98.2% of the total mutations (Tables S2 and S3).

## 4. Discussion

This study showed a high diagnostic yield with a universal, sequential active screening of FH index cases. The prevalence of FH within the studied cohort of patients with severe HCL was 12.2%, with a genetically confirmed FH rate in the DLCN ≥ 6 group of 67.9%. We estimate that the strategy proposed in this study will contribute to modifying how FH index case screening is conducted because in our health setting, where only 36.8% of the patients with FH mutation had been previously diagnosed, the FH detection increased 2.3 times.

Our screening strategy presents several advantages that might justify the higher detection rates obtained. It focuses on patients with a higher probability of having FH by clinical diagnosis (DLCN ≥ 6), since this is the group that is really considered FH [10] and, therefore, are candidates for advanced lipid-lowering therapies in accordance with the recommendations of Spanish guidelines [7,30]. Additionally, it allows optimization of diagnostic yield in accordance with the available resources. This is why, unlike cascade screening, potential index cases with DLCN 3–5 were not genetically tested, except for 10 patients, to estimate the diagnostic yield of our study (20%), in a similar way as previously described [31,32,33].

The first step in the search for patients was to detect those who had high LDL-C levels using widely available tools such as the computerized, centralized analytical data of the population in the health area obtained from both primary and hospital care. The exclusive use of LDL-C levels for the FH screening may be debatable, as not all patients with genetic mutations express the HCL phenotype [10]. Nevertheless, it is the most useful tool for initial screening because it is the most characteristic and frequent phenotype abnormality, particularly among the young [34], it is easily recognizable by any clinician, and it is the indicator that best predicts a later positive diagnosis of FH [9,35]. The advantage of this study was supplementing the initial analytical screening with a direct medical assessment, which increased diagnostic accuracy with a concise check of the items included in the DLCN criteria. In fact, 83.3% of the genetically tested patients had a family member with a history of hypercholesterolemia that would score points on the DLCN, data that might easily be omitted from digital medical records.

Recent studies have evaluated the genetic yield of FH with NGS. Reeskamp et al. [36] reported an overall prevalence of genetically confirmed FH of 14.9% within a cohort of 1528 patients with clinical FH diagnosis at a national referral center for genetic diagnosis. The FH detection rate in subjects with DLCN ≥ 6, once stratified by higher LDL-C levels and stricter diagnostic FH criteria, was more than 50%. The higher prevalence in this study may be related to including subjects with DLCN 3–5 for genetic testing. Our better diagnosis yield in the DLCN ≥ 6 group could be because our cohort exhibited higher mean LDL-C levels, fewer missing patient data for DLCN calculation, and restricted access to genetic testing. In Wang et al. [37], among 313 patients with severe HCL (LDL-C ≥ 190 mg/dL), 65.5% of them with DLCN ≥ 6, a FH causative mutation was identified in 148 (47.3%). The detection rate increased up to 88% in those with LDL-C ≥ 310 mg/dL. Trinder et al. [38] found a pathogenic FH variant in 275 of 626 patients (43.9%) with previous clinical FH diagnosis who were referred for NGS. Of their cohort, 456 patients (72.8%) had a probable or definite clinical FH diagnosis. The percentage of genetic confirmation in patients with DLCN 6–7 and DLCN ≥ 8 was 37.4% and 74.3%, respectively. We reported a lower prevalence of genetically confirmed FH compared to these two studies. However, only 17.9% of our cohort had DLCN ≥ 6, and subjects with possible FH were not genetically tested (except for 10). Despite this, in our study, the LDL-C level was the most weighted item of the DLCN criteria, and the greatest diagnosis rate was in DLCN ≥ 8, as in Wang et al., where the higher the LDL-C level, the higher the diagnostic yield. Our higher percentage of genetic diagnosis in both the DLCN 6–7 and DLCN ≥ 8 groups, compared to Trinder et al., could be explained because the same professional who applied the DLCN criteria also obtained the samples for genetic analysis. Consequently, there might have been stricter patient classification.

Other screening strategies for FH have previously been studied, both among general and selected populations. Khera et al. [15] studied a population with severe HCL without ASCVD and obtained a 2% prevalence of FH. The difference in the results may be explained because the definition of severe HCL was LDL-C ≥ 190 mg/dL, with the consequent loss of specificity compared to our cut-off point of LDL-C ≥ 220 mg/dL. In addition, this study did not include patients with ASCVD, and there was no direct medical assessment. Benn et al. [2] reported a prevalence of 5% of FH in subjects with LDL-C ≥ 220 mg/dL, conducted using a large cohort of the general Danish population. The best diagnostic yield was obtained in patients with LDL-C ≥ 230 mg/dL (13%). Nevertheless, the percentage of genetic diagnosis of FH in DLCN 6–7 and ≥8 points was 6% and 24%, respectively, whereas our study showed 58.9% and 85.7%, respectively. This result may be due to only having analyzed four genetic variations and not performing a direct medical assessment of the patients, therefore omitting important data. However, our study represents a selected population sample, which might explain the difference in FH prevalence compared to the two previous studies. Abul-Husn et al. [39] described a similar situation in their randomized cohort of patients whose digital medical records they analyzed to perform a retrospective diagnosis of FH according to DLCN criteria. Of their cohort, 10% had the severe HCL phenotype, with 2.5% prevalence of FH and 12.8% of genetic diagnosis in the group with LDL-C ≥ 250 mg/dL. Finally, in Amor-Salamanca et al., the prevalence of genetically confirmed FH was 9% in those cases ≤65 years admitted for ACS and with LDL-C ≥ 160 mg/dL [28]. Nonetheless, index cases were detected after the development of clinical ASCVD; therefore, following such a strategy does not allow the identification of asymptomatic FH patients.

As previously stated, this study confirms that the diagnosis of index cases continues to be random [1,25] and clearly improvable [20]. Given the prognostic significance of early detection and treatment both for FH index cases and relatives, we consider it necessary to apply screening strategies at the general population level. The screening strategy presented, which focuses on the search for patients with a higher probability of having FH, increases diagnostic accuracy and allows subsequent family-based cascade screening, in addition to an efficient and rational use of healthcare resources [40].

One of the notable limitations of our study is that it was not conducted among the general population, but rather in a severe HCL population. The objective of this study was to establish a realistic scenario, which is why we focused on subjects with a higher probability of having FH. Self-selection bias may exist in patients with previous ASCVD as well as family history of hypercholesterolemia and/or premature ASCVD. In addition, selection bias may have existed with the patient selection strategy and may have influenced the final result due to the deliberate exclusion of patients with an apparent secondary cause of hypercholesterolemia who could have FH. It is also possible that this strategy may have allowed subjects with FH and not such abnormally high LDL-C to go unnoticed, as well as those with a clinical possible FH diagnosis, although it was estimated that this represents a small number of the adult population [10], the majority of whom can be identified in a family-based cascade screening. Likewise, due to financial restrictions, it was not possible to conduct genetic testing of all the medically assessed patients, although we indicate that a large number of tests was performed. Finally, it was not possible to analyze some data from the medical records of relatives of index cases, such as the presence of tendon xanthoma, nor data related to non-resident relatives in the health area studied.

## 5. Conclusions

In conclusion, after applying a strategy of active, sequential screening of index cases, the prevalence of FH in the severe HCL cohort of patients was 12.2%, with a high percentage of genetically confirmed FH among the target population (67.9%). It is essential to accurately select patients who should undergo genetic testing based on centralized analytical data for a subsequent medical assessment, which can reliably stratify the patients who have suspected FH. Our data support the applicability of an active screening strategy at the general population level, with a rational use of the available resources, which would facilitate the early detection and treatment of FH index cases and the application of family-based cascade screening.

## Figures and Tables

**Table 1:** Baseline characteristics.

Variable	Overall (*n* = 469)	DLCN 3–5 (*n* = 385)	DLCN ≥ 6 (*n* = 84)	Standardized Effect Size (95% CI)
Male	191 (40.7)	162 (42.1)	29 (34.5)	0,73 (0.44 to 1.19)
Mean age, years	53.2 ± 12.8	54.6 ± 12.3	47.1 ± 12.9	0.59 (0.36 to 0.82) *
Hypertension	148 (31.6)	130 (33.8)	18 (21.4)	0.54 (0.31 to 0.94) *
Diabetes	47 (10.0)	43 (11.2)	4 (4.8)	0.39 (0.14 to 1.14)
Current or past smoking	233 (49.7)	181 (47.0)	52 (61.9)	1.83 (1.13 to 2.97) *
Body Mass Index	27.7 ± 4.4	27.8 ± 4.3	27.0 ± 4.7	0.19 (−0.04 to 0.43)
ASCVD	26 (5.5)	13 (3.4)	13 (15.5)	5.24 (2.33 to 11.77) *
Premature ASCVD	11 (2.3)	3 (0.8)	8 (9.5)	13.4 (3.48 to 51.68) *
Family history of premature ASCVD	34 (7.2)	15 (3.9)	19 (22.6)	7.21 (3.49 to 14.91) *
Family history of HCL	131 (27.9)	61 (15.9)	70 (83.3)	26.48 (14.02 to 49.99) *
Corneal arcus (<45 years)	7 (6.2)	0	7 (20.6)	-
Tendon Xanthoma	1 (0.2)	0	1 (1.1)	-
Total cholesterol (mg/dL)	331.7 ± 48.3	317.7 ± 21.03	396.2 ± 77.5	−1.62 (−1.97 to −1.27)
LDL-C (mg/dL)	246.8 ± 38.2	234.9 ± 15.6	301.5 ± 58.5	−1.74 (−2.08 to −1.41)
HDL-C (mg/dL)	56.2 ± 14.0	56.4 ± 13.6	55.2 ± 15.9	0.89 (−1.47 to 0.33)
Triglycerides (mg/dl)	128.3 ± 36.3	129.5 ± 35.8	122.8 ± 38.4	0.18 (−0.05 to 0.42)
TSH (mLU/L)	2.1 ± 2.12	2.1 ± 2.3	2.2 ± 1.30	−0.08 (−0.32 to 0.17)
Lipid-lowering treatment	344 (73.4)	269 (69.9)	75 (89.3)	3.59 (1.74 to 7.42) *
Maximal lipid-lowering therapy	109 (23.2)	64 (16.6)	45 (53.6)	5.79 (3.49 to 9.59) *

Values are mean ± standard deviation (SD) or *n* (%). * *p* < 0,05. ASCVD: atherosclerotic cardiovascular disease. CI: Confidence interval. DLCN: Dutch Lipid Clinic Network. HCL: Hypercholesterolemia. HDL-C: high-density lipoprotein cholesterol. LDL-C: low-density lipoprotein cholesterol. TSH: Thyroid stimulating hormone.

**Table 2:** Atherosclerotic cardiovascular disease rates in DLCN ≥ 6.

	DLCN ≥ 6 no FH Mutation (*n* = 27)	DLCN ≥ 6 FH Mutation (*n* = 57)	*p*-Value
ASCVD	2 (7.4)	11 (19.3)	0.33
ASCVD without previous FH diagnosis	2 (100)	7 (63.6)	0.47
Premature ASCVD	1 (50)	7 (63,6)	0.43
Premature ASCVD without previous FH diagnosis	1 (100)	3 (42.9)	1
Coronary artery disease	2 (100)	7 (63.6)	0.72
Cerebral vascular disease	0	4 (36.4)	-
Peripheral vascular disease	1 (50)	1 (9.1)	0.51

Values are *n* (%). FH: Familial hypercholesterolemia. Other abbreviations as in Table 1.

**Table 3:** Genetic testing results.

	All	FH Mutation	No FH Mutation	Odds Ratio (95% CI)	*p*-Value *
Previous FH diagnosis	26	21 (80.8)	5 (19.2)	2,94 (0.99–8.73)	0.08
Possible FH	10	2 (20)	8 (80)	1 (Reference)	-
Probable FH	56	33 (58.9)	23 (41.1)	5.74 (1.12–29.54)	0.037
Definite FH	28	24 (85.7)	4 (14.3)	24.0 (3.68–156.7)	0.001
DLCN ≥ 6	84	57 (67.9)	27 (32.1)	8.44 (1.68–42.49)	0.005

Values are *n* (%). * *p* < 0.05. ** Percentages according to no FH mutation of the respective DLCN group. Abbreviations as in Table 1 and Table 2.

## Data Availability

Data is contained within the article and supplementary Materials.

## Supplementary Materials

The following are available online at https://www.mdpi.com/2077-0383/10/4/749/s1, Table S1: Dutch Lipid Clinic Network criteria, Table S2. Causative mutations in DLCN ≥ 6 group, Table S3: Genetic variants in DLCN ≥ 6 group.
