# Supplementary material for: Improving Familial Hypercholesterolemia Index Case Detection: Sequential Active Screening from Centralized Analytical Data
Source: J Clin Med. 2021 Feb 13;10(4):749. doi: 10.3390/jcm10040749 (PMC7918446; doi:10.3390/jcm10040749)
Supplement: Supplementary file 1 [file jcm-10-00749-s001.pdf]

SUPPLEMENTARY MATERIAL

Table S1: Dutch Lipid Clinic Network criteria

	Points
**Family history**	
First-degree relative with premature ASCVD<br>OR<br>First-degree relative with LDL-C ≥ 210 mg/dl	1<br><br>1
First-degree relative with corneal arcus and/or tendon xanthoma<br>OR<br>Children < 18 years with LDL-C ≥ 150 mg/dl	2<br><br>2
**Clinical history**	
Patient with premature coronary artery disease	2
Patient with premature cerebral or peripheral vascular disease	1
**Physical examination**	
Tendon xanthoma	6
Corneal arcus < 45 years	4
**Analytical results**	
LDL-C ≥ 330 mg/dl	8
LDL-C ≥ 250 - 329 mg/dl	5
LDL-C ≥ 190 - 249 mg/dl	3
LDL-C ≥ 155 - 189 mg/dl	1
**Genetic testing**	
Causative mutation in the *LDLR*, *APOB* or *PCSK9* genes	8
**FH definite: ≥ 8 points Probable FH: 6-7 points**<br>**FH possible: 3-5 points Unlikely FH: ≤ 2 points**	

Table S2: Causative mutations in DLCN ≥ 6 group

**Positive genetic study**	57 (100)
• ***LDLR*** **gene**	53 (92.9)
• ***APOB*** **gene**	3 (5.3)
• ***APOE*** **gene**	1 (1.8)
• ***LDLR*** **or** ***APOB*** **gene**	56 (98.2)

Table S3: Genetic variants in DLCN ≥ 6 group

Patient	Sex	Age	DLCN	Genetic variant	Variant classification
1	Female	35	12	*LDLR* c.1342C>T p.(Gln427*)	Pathogenic
2	Male	69	6	*LDLR* c.1342C>T p.(Gln427*)	Pathogenic
3	Female	49	10	*LDLR* c.1898G>A, p.(Arg612His)	Pathogenic
4	Female	56	9	*LDLR* c.1238C>T, p.(Thr413Met)	Likely pathogenic
5	Female	60	6	*LDLR* c.622G>A, p.(Glu208Lys)	Likely pathogenic
6	Male	47	9	*LDLR* c.126C>A, p.(Tyr42*)	Pathogenic
7	Female	34	9	*LDLR* c.1261A>G, p.(Ser421Gly)	Likely pathogenic
8	Male	40	11	*LDLR* c.1587-2A>G, p.(?)	Pathogenic
9	Male	29	9	*LDLR* c.676T>C, p.(Ser226Pro)	Pathogenic
10	Female	83	9	*LDLR* c.1054T>C, p.(Cys352Arg)	Likely pathogenic
11	Female	27	6	*LDLR* c.313+1G>C, p.(?)	Pathogenic
12	Female	50	9	*LDLR* c.313+1G>C, p.(?)	Pathogenic
13	Female	35	6	*LDLR* c.1775G>A, p.(Gly592Glu)	Pathogenic
14	Male	75	8	*LDLR* c.1925T>C, p.(Leu642Ser)	Likely pathogenic
15	Female	59	9	*LDLR* c.1342C>T, p.(Gln448*)	Pathogenic
16	Female	32	6	*LDLR* c.1342C>T, p.(Gln448*)	Pathogenic
17	Female	53	6	*LDLR* c.2546C>A, p.(Ser849*)	Pathogenic
18	Female	34	6	*LDLR* c.967G>A, p.(Gly323Ser)	Likely pathogenic
19	Female	36	9	*LDLR* c.47T>C, p.(Leu16Pro)	Likely pathogenic
20	Female	30	7	*LDLR* c.1444G>A, p.(Asp482Asn)	Likely pathogenic
21	Male	49	6	*APOB* c.13151T>C, p.(Leu4384Pro)	Likely pathogenic
22	Female	66	6	*LDLR* c.1246C>T, p.(Arg416Trp)	Pathogenic
23	Female	47	7	*LDLR* c.1981C>A, p.(Pro661Thr)	Likely pathogenic
24	Female	43	7	*LDLR* c.1981C>A, p.(Pro661Thr)	Likely pathogenic
25	Male	32	9	*LDLR* c.1342C>T, p.(Gln448*)	Pathogenic
26	Female	51	6	*LDLR* c.1054T>C, p.(Cys352Arg)	Likely pathogenic
27	Female	65	6	*LDLR* c.1246C>T, p.(Arg416Trp)	Pathogenic
28	Male	55	7	*LDLR* c.530C>T, p.(Ser177Leu),	Pathogenic
29	Male	46	7	*LDLR* c.2099A>G, p.(Asp700Gly)	Likely pathogenic
30	Female	48	6	*LDLR* c.593C>A, p.(Ser198*)	Pathogenic
31	Female	25	6	*LDLR* c.337G>A, p.(Glu113Lys)	Likely pathogenic
32	Female	53	7	*APOE* c.500_502del, p.(Leu167del)	Pathogenic
33	Male	34	11	*LDLR* c.1055_1060+5del, p.(?)	Pathogenic
34	Female	61	9	*LDLR* c.2275_2302del, p.(Thr761Lysfs*18)	Pathogenic
35	Male	32	6	*LDLR* c.2275_2302del, (p.(Thr761Lysfs*18)	Pathogenic
36	Male	33	6	*LDLR* c.1618G>A, p.(Ala540Thr)	Likely pathogenic
37	Male	41	6	*LDLR* c.1618G>A, p.(Ala540Thr)	Likely pathogenic

38	Male	56	6	*LDLR* c.1216C>T, p.(Arg406Trp)	Likely pathogenic
39	Male	58	7	*LDLR* c.2099A>G, p.(Asp700Gly)	Likely pathogenic
40	Female	37	7	*LDLR* c.2099A>G, p.(Asp700Gly)	Likely pathogenic
41	Male	37	10	*LDLR* c.1342C>T, p.(Gln448*),	Pathogenic
42	Male	47	6	*LDLR* c.1216C>T, p.(Arg406Trp)	Likely pathogenic
43	Female	38	9	*LDLR* c.1434del, p.(Leu479Trpfs*28)	Pathogenic
44	Female	35	6	*LDLR* c.81C>G, p.(Cys27Trp)	Pathogenic
45	Female	58	6	*APOB* c.10580G>A, p.(Arg3527Gln)	Pathogenic
46	Female	53	6	*APOB* c.13151T>C, p.(Leu4384Pro)	Likely pathogenic
47	Female	31	11	*LDLR* c.1342C>T, p.(Gln448*)	Pathogenic
48	Female	57	6	*LDLR* c.1342C>T, p.(Gln448*)	Pathogenic
49	Male	56	7	*LDLR* c.1618G>A, p.(Ala540Thr)	Likely pathogenic
50	Male	37	10	*LDLR* c.313+1G>C	Pathogenic
51	Male	27	6	*LDLR* c.1601C>A, p.(Thr534Asn)	Likely pathogenic
52	Male	45	11	*LDLR* c.1775G>A, p.(Gly592Glu)	Pathogenic
53	Female	49	6	*LDLR* c.530C>T, p.(Ser177Leu)	Pathogenic
54	Female	45	9	*LDLR* c.2140+1G>T	Pathogenic
55	Male	36	13	*LDLR* c.1342C>T, p.(Gln448*)	Pathogenic
56	Female	39	9	*LDLR* c.313+1G>C	Pathogenic
57	Female	42	13	*LDLR* c.1061-?_1586+?del	Pathogenic
58	Male	46	5	*LDLR* c.2475C>A, p.(Asn825Lys)	Pathogenic
59	Female	55	4	*APOB* c.6639_6641del, p.(Asp2213del)	Likely pathogenic
